# Immune and Endothelial-Related Extracellular Vesicles Are Associated with Corticosteroid Response and Mortality in Alcohol-Associated Hepatitis

**DOI:** 10.3390/ijms27031258

**Published:** 2026-01-27

**Authors:** Albert Guinart-Cuadra, Anna Brujats, Justyna Szafranska, Rubén Guerrero, Fernándo Dinamarca, Elisabet Cantó, Maria Poca, Eva Román, Elisabet Sánchez-Ardid, Javier Fajardo, Montserrat Camps, Maria Mulet, German Soriano, Àngels Escorsell, Juan M. Falcon-Perez, Esperanza Gonzalez, Andreu Ferrero-Gregori, Cristina Gely, Jorge Villalba, Ramón Bataller, Josepmaria Argemi, Rubén Osuna-Gómez, Silvia Vidal, Edilmar Alvarado-Tapias

**Affiliations:** 1 Group of Inflammatory Diseases, Institut de Recerca Sant Pau (IR SANT PAU), 08041 Barcelona, Spain; aguinart@santpau.cat (A.G.-C.); rosuna@santpau.cat (R.O.-G.); svidal@santpau.cat (S.V.); 2 Centre for Biomedical Research in Liver and Digestive Diseases Network (CIBERehd), Instituto de Salud Carlos III, 28029 Madrid, Spain; abrujats@santpau.cat (A.B.);; 3 Departament de Biologia Cellular, Fisiologia i Immunologia, Universitat Autònoma de Barcelona, Campus UAB, 08193 Cerdanyola del Vallès, Spain; 4 Gastroenterology and Hepatology Department, Hospital de la Santa Creu i Sant Pau, Department of Medicine, Universitat Autònoma de Barcelona, 08025 Barcelona, Spain; 5 Department of Pathology, Hospital de la Santa Creu i Sant Pau, 08041 Barcelona, Spain; 6 Department of Interventional Radiology, Hospital de la Santa Creu i Sant Pau, 08041 Barcelona, Spain; 7 Department of Psychiatry, Hospital de la Santa Creu i Sant Pau, Institut d’Investigació Biomèdica Sant Pau (IIB SANT PAU), 08041 Barcelona, Spain; 8 Exosomes Laboratory, Center for Cooperative Research in Biosciences (CIC bioGUNE), Basque Research and Technology Alliance (BRTA), 48160 Elaxalde Derio, Bizkaia, Spain; 9 Ikerbasque, Basque Foundation for Science, 48009 Bilbao, Spain; 10 Liver Unit, Department of Digestive and Metabolic Diseases, Hospital Clínic de Barcelona, 08036 Barcelona, Spain; 11 Liver Unit and HPB Oncology Area, Clinica Universidad de Navarra, 31008 Pamplona, Spain; 12 Computational Biology and Translational Genomics Program, Center for Applied Medical Research (CIMA), University of Navarra, 31008 Pamplona, Spain; 13 Instituto de Investigación Sanitaria de Navarra (IdiSNA), 31008 Pamplona, Spain; 14 Solid Tumors Program, Hepatology Laboratory, Center for Applied Medical Research (CIMA), University of Navarra, 31009 Pamplona, Spain

**Keywords:** alcohol-associated hepatitis, extracellular vesicles, corticosteroid response

## Abstract

Alcohol-associated hepatitis (AH) is the most severe clinical manifestation of alcohol-associated liver disease. Corticosteroids are the only disease-specific therapy shown to improve short-term survival. Currently, no non-invasive markers are available to predict patient response to corticosteroids or long-term survival in AH. This study investigates whether surface antigens on plasma extracellular vesicles (EVs), key mediators of intercellular communication, can reflect the underlying immune dysregulation in AH and serve as prognostic markers. Patients with AH were prospectively enrolled between 2020 and 2024. Blood samples were collected before corticosteroid initiation during the first 24 h of hospitalization. EVs were characterized using nanoparticle tracking analysis, cryo-electron microscopy, and flow cytometry. Interleukin-6 (IL-6), soluble (s)CD62p, Circulating Vascular Cell Adhesion Molecule-1 (sVCAM), tumor necrosis factor receptor superfamily member 1 (TNRFS1a), and Intercellular Adhesion Molecule 1 (ICAM-1) were quantified by ELISA. Key outcome variables included response to corticosteroids and mortality. A total of 46 patients with AH and 28 healthy donors (HD) were included. EV concentration was significantly higher in AH patients than in HD (9.3 × 10^11^ [IQR 4–24] versus 2.4 × 10^11^ [IQR 2–4], *p* = 0.03). Specific EV antigens were associated with key clinical outcomes: CD20 and CD2 levels differed between patients with or without infections (bacterial, viral, and fungal) developed during hospitalization; CD40 and CD146 were elevated in patients who developed acute kidney injury. EVs enriched in monocyte (CD14) and T-reg (CD25) markers were associated with plasma IL-6 levels, while endothelial markers CD105 and CD146 correlated with sVCAM and sCD62p. EVs enriched in platelet (CD49e) and endothelial (CD31) markers were associated with corticosteroid response, whereas EVs enriched with endothelial (CD105 and CD146) and B lymphocyte (CD19) markers were associated with mortality. Overall, EVs enriched in endothelial and monocyte markers may represent a candidate non-invasive tool for predicting corticosteroid response and mortality in AH, aiding risk stratification and early identification of non-responders for timely transplant evaluation.

## 1. Introduction

Alcohol-associated liver disease (ALD) is the leading cause of advanced liver disease worldwide [1,2]. Alcohol-associated hepatitis (AH) is the most severe and complex manifestation of ALD. It is associated with systemic inflammatory response syndrome (SIRS) [3], immune dysfunction (i.e., the dysregulation of many cells, subpopulations, and the suppression of both innate and adaptive immune responses in liver cells) [4,5], favoring bacterial infections [6], and acute kidney injury (AKI) [7]. A third of AH patients develop acute-on-chronic liver failure [8], a condition with high short-term mortality (20 to 50% at three months) [9]. Abstinence remains the cornerstone of treatment, although alcohol relapse is frequent after an episode of AH [10]. Corticosteroids are currently the only disease-specific therapy shown to improve short-term survival, within 28 days, in selected patients [11,12,13]. However, their use further amplifies the underlying immune dysfunction and is associated with an increased risk of severe infections, which significantly impacts prognosis and limits their clinical benefit [14,15]. Therefore, careful patient selection is critical to balance potential survival benefits against the heightened risk of infectious complications [16,17,18]. Currently, no non-invasive biomarkers are available to reliably predict patient response to corticosteroids or long-term survival in AH. Moreover, several novel therapeutic strategies have been explored, including modulation of inflammatory pathways, targeting gut–liver axis dysfunction, antioxidant therapies, and regenerative or cell-based approaches; however, none have demonstrated consistent clinical benefit to date [19].

AH is marked by complex immune activation, including neutrophil, monocyte/macrophage, and T-cell recruitment, cytokine storm, and paradoxical immunosuppression [5]. Concurrently, elevated levels of endothelial dysfunction markers (Vascular Cell Adhesion Molecule 1 (VCAM-1), Intercellular Adhesion Molecule 1 (ICAM-1), Cluster of Differentiation 146 (also known as MCAM-CD146), and Vascular Endothelial Growth Factor A (VEGF-A) correlate with disease severity, portal hypertension, and mortality [20]. Notably, these markers remain persistently elevated for up to 12 months, even after alcohol abstinence, indicating sustained endothelial injury [21]. However, chronic endothelial activation has not yet been recognized as a contributing mechanism to the persistent immune dysfunction observed after an episode of AH. Advancing effective therapies in AH will require a deeper understanding of the cellular crosstalk between immune cells and endothelial dysfunction that underpins AH and its clinical complications.

Promising, yet less investigated markers in ALD are extracellular vesicles (EVs) [22,23]. EVs are nano-sized membrane-bound particles, encompassing small and large vesicle populations, released by cells under physiological or pathological conditions and carrying cargo that reflects their cellular origin and functional state [24,25]. EVs, which are found in biological fluids, may serve as circulating biomarkers reflecting immune and endothelial alterations rather than as direct mediators of disease pathogenesis, while also participating in cell-to-cell communication [24,26]. The release of EVs increased, and their composition differs in ALD and AH patients compared to healthy individuals [16,27,28]. EVs may modify transcription in neighboring hepatocytes and non-parenchymal cells, modulating liver inflammation and fibrosis [27,28,29,30]. EVs in AH are enriched with specific microRNAs, especially miR192, miR122, and miR30a, with miR192 showing the strongest diagnostic potential [29]. Further studies have documented that EVs also carry damage-associated molecular patterns (DAMPs), activate macrophages via TLR9, and upregulate pro-inflammatory cytokine production (e.g., IL-1β and TNFα), suggesting that EVs may consequently play a crucial role in cellular crosstalk during an AH episode [28,29,31].

However, it remains unknown whether the surface antigen of circulating EVs, particularly the enrichment of EVs with immune and endothelial markers, can serve as non-invasive indicators of the interplay between immune dysregulation and endothelial dysfunction in AH. Specifically, the potential association between EV enrichment in selected surface antigens and clinically relevant outcomes, such as response to corticosteroid therapy, and short- and long-term prognosis, has not yet been established.

Therefore, the aim of this study was to characterize the enrichment of immune- and endothelial-associated surface antigens on plasma EVs in patients with AH, and to evaluate their association with immune–endothelial crosstalk, corticosteroid response, short- and long-term clinical outcomes.

## 2. Results

During the study period, 58 patients with a clinical suspicion of AH were admitted to Hospital de la Santa Creu i Sant Pau. Among them, 46 patients were included in the study (Supplementary Figure S1). The epidemiological, clinical, laboratory, and hemodynamic characteristics of the included patients are shown in Table 1. Patients with AH were predominantly young males, with 76% reporting tobacco use and 35% presenting features of metabolic syndrome. For 76% of patients, this was their first admission, with ascites as the initial decompensation in 41%. Of the patients included, 76% had biopsy-confirmed AH. The Model for End-Stage Liver Disease (MELD), the Maddrey DF score, and ABIC scores were similar regardless of histological confirmation. Hemodynamic assessments were performed on 87% of the cohort.

### 2.1. Patients with AH Had Increased Numbers of Circulating EVs with a Different EV Antigen Profile Compared to Healthy Donors

A quantitative characterization by Nanoparticle Tracking Analysis (NTA) showed a significantly increased size heterogeneity (Supplementary Figure S2A,B) and total number (Supplementary Figure S2C) of circulating EVs in the plasma of AH patients compared to HD (particles/mL 9.3 × 10^11^ (IQR 4–24) versus 2.4 × 10^11^ (IQR 2–4), *p* = 0.03). The majority of the EVs identified in the two groups ranged in size from 40 to 200 nm, with an increased prevalence of larger and more size-disperse EVs in the plasma samples of AH patients when compared to HD (Supplementary Figure S2A,B). When observed by Cryo-electron microscopy (CryoEM), EVs had a characteristic spherical or cup-shaped morphology (Supplementary Figure S2D).

**Figure 1:**
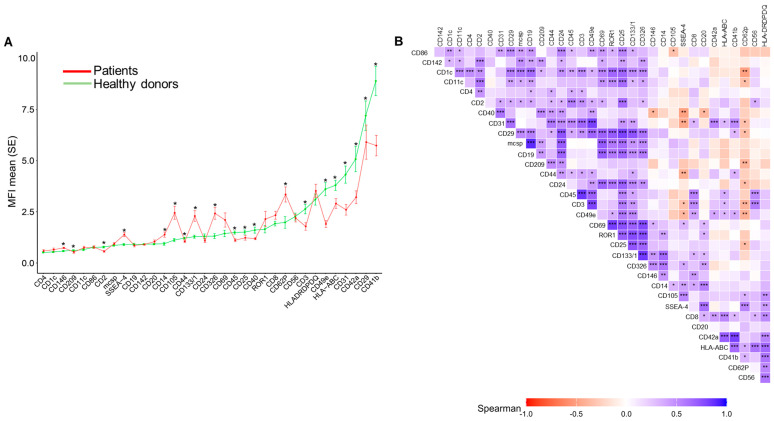
Surface antigen profiles of extracellular vesicles (EVs) in healthy donors (HD) and alcohol-associated hepatitis (AH) patients: (**A**) Mean fluorescence intensity (MFI) profiles of 33 surface antigens expressed on EVs from HD (*n*= 28, green) and patients with AH (*n* = 46, red). Data are presented as mean ± standard deviation. Differences between groups were assessed using *t*-tests or Mann–Whitney tests, depending on the distribution of the values. (**B**) Correlation matrix of surface antigens expressed in EVs from AH patients. The heatmap displays pairwise Spearman correlation coefficients, with positive correlations shown in blue and negative correlations in red. * *p* < 0.05, ** *p* < 0.01, *** *p* < 0.001.

Plasma EVs surface was phenotyped by flow cytometry using a MACSPLEX EV kit IO (Miltenyi, Bergisch Gladbach, Germany), human with 37 surface antigens. Among them, 20 were differentially expressed between the two groups (Figure 1A, Supplementary Table S1). Thirteen antigens were significantly lower in EVs from AH patients. These included cell-specific antigens from leucocytes (CD45), T-cells (CD2, CD3, and CD25), monocyte-macrophages (CD44, CD40, CD209, and CD29) and platelets (CD41b and CD42a), and specific functional markers such as the integrin of monocytes-platelets (CD49e), cell adhesion molecule required for leukocyte transendothelial migration (CD31), and the antigen-presenting molecule (HLA-ABC). In AH patients, there was a significantly higher proportion of EVs enriched with seven endothelial cell antigens (CD105, CD146, and CD62p), followed by epithelial (CD133 and CD326), stem cell (SSEA-4), and monocyte-macrophage (CD14) lineages (Figure 1A). A correlation analysis of EV antigens revealed conserved positive associations between the two groups but disrupted negative correlations in AH, but not in HD (Figure 1B and Supplementary Figure S3).

### 2.2. EVs Surface Antigens Are Associated with the Main Clinical Features in the AH Episode

**Figure 2:**
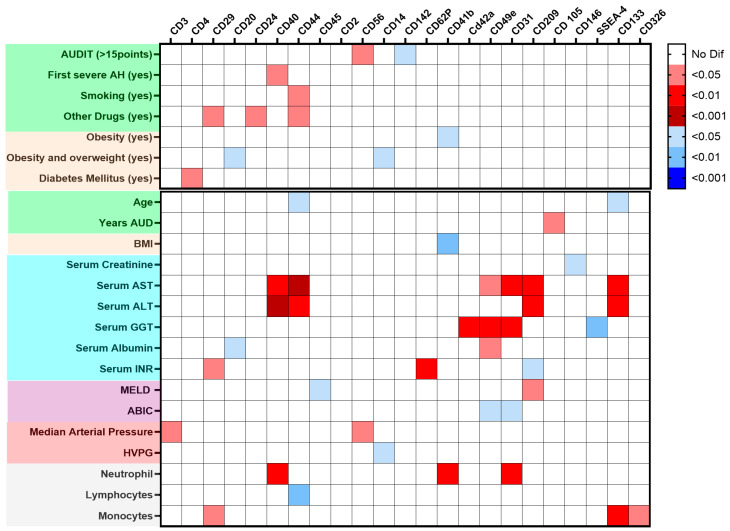
Heatmap of the main extracellular vesicles (EVs), EV surface antigen, and the clinical data of alcohol-associated hepatitis (AH) patients. Data are presented as a heatmap built considering the *p*-value for *p*-values comparing the main EV surface antigens between patients (*n* = 46) and healthy donors HD (*n* = 28), along with qualitative and quantitative clinical characteristics at admission, colors on the left of both panels indicate feature types: green, age and toxic substance use; beige, weight and metabolic disorders; blue, biochemical values; purple, prognostic indices; red, hemodynamics parameters; and gray, blood cell counts. **Top panel:** Mann–Whitney tests were performed to evaluate differences in surface antigen expression in dichotomous clinical variables. The intensity represents the level of significance, with red indicating positive associations and blue indicating negative associations. **Bottom panel:** Correlation analysis between EV surface marker expression and continuous clinical variables. Spearman’s rank correlation coefficients were used, with red indicating positive correlations and blue indicating negative correlations.

Figure 2 shows a graphical analysis that linked EVs surface antigens with clinical and epidemiological features in AH patients. The Alcohol Use Disorder Identification Test (AUDIT) score value, age, and drug use were associated with increased EVs from endothelial and progenitor cells and decreased EVs from immune cells. In contrast, metabolic risk factors (diabetes mellitus, body mass index [BMI], overweight, and obesity) correlated with increased EVs from platelets, endothelium, and monocytes. Specific blood cell counts also showed distinct correlations with EV profiles.

Renal and liver function parameters showed distinct correlations with EV profiles. Creatinine levels were inversely correlated with leukocyte and EVs expressing endothelial markers. The transaminases aspartate aminotransferase (AST) and alanine aminotransferase (ALT) were positively correlated with EVs expressing monocyte, endothelial, and progenitor cell markers, whereas gamma-glutamyl transferase (GGT) correlated with platelet EVs and EVs expressing endothelial markers. Total bilirubin and albumin were negatively associated with EVs expressing endothelial markers, whereas international normalized ratio (INR) was positively linked to endothelial EVs and negatively linked to EVs expressing monocyte markers. Hemodynamically, arterial pressure was positively associated with EVs expressing lymphocyte markers, and hepatic venous pressure gradient (HVPG) was inversely correlated with EVs expressing monocyte markers (Figure 2).

EVs’ surface antigens were correlated with liver function scores at admission (Supplementary Figure S4. The MELD score showed a positive correlation with EVs expressing monocyte markers (CD209) and a negative correlation with EVs expressing leukocyte markers (CD45). The ABIC score was inversely associated with monocyte-platelet integrin antigens (CD49e) and leukocyte transmigration (CD31). The Child–Pugh score negatively correlated with EVs expressing T cell (CD2, CD3), leukocyte (CD45), platelet integrin (CD49e), platelet antigen (CD42a), leukocyte transmigration (CD31), and activation/adhesion antigens (CD25, CD29).

In patients with AH, the most frequent complication during the index hospitalization was infection, occurring in 20 patients (43%). A total of 30 infectious episodes were identified, including bacterial pneumonia (6.21%), bacteremia (6.21%), urinary tract infection (6.21%), culture-negative spontaneous bacterial peritonitis (4.13%), esophageal candidiasis (2.7%), *Clostridioides difficile* colitis (2.7%), cellulitis (2.7%), and viral infections (2.7%). Another frequent complication was the AKI, observed in 12 patients (26%) (Supplementary Table S2).

**Figure 3:**
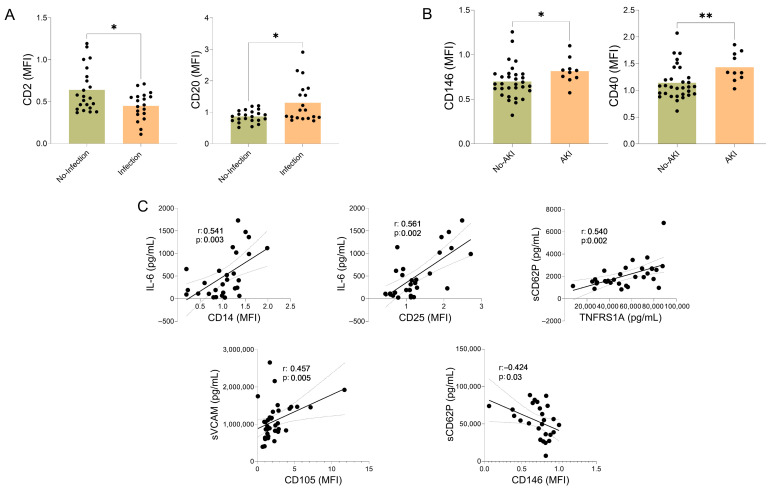
Antigens on the surface of extracellular vesicles (EVs), infection, acute kidney injury (AKI), and soluble inflammatory mediators in AH patients. Association of EV surface antigens with infection, acute kidney injury (AKI), and soluble inflammatory mediators in AH patients. (**A**) Expression of CD2 and CD20 on plasma-derived EVs in AH patients with and without infections, data are presented as mean ± standard deviation. Differences between groups were assessed using *t*-tests or Mann–Whitney tests, depending on the distribution of the values; * *p* < 0.05. (**B**) Expression of CD146 and CD40 on plasma-derived EVs in AH patients with and without AKI, * *p* < 0.05, ** *p* < 0.01. The black dots indicate the values for each patient individually. (**C**) Correlation analysis between EV surface antigen expression and circulating soluble inflammatory mediators. Spearman’s correlation coefficient (r) and corresponding *p*-values are shown. The gray lines indicate the confidence intervals.

Among patients who developed infections during hospitalization, we observed a higher proportion of EVs expressing B-cell markers (CD20) and a lower proportion expressing T-cell markers (CD2). In contrast, patients with AKI showed increased levels of EVs expressing monocyte antigen-presenting markers (CD40) and endothelial markers (CD146). In addition, several soluble inflammatory markers, including IL-6, sVCAM-1, sCD62P, and tumor necrosis factor receptor superfamily member 1 (TNFRSF1A), were correlated with EVs expressing monocyte and activated cell markers (CD14, CD25), as well as with EVs expressing endothelial markers (CD105 and CD146) (Figure 3).

### 2.3. Diagnostic Performance of the EV Surface Antigen Associated with Corticosteroid Response and Long-Term Mortality

This study assessed whether EVs could be associated with corticosteroid response and survival in patients with AH. Among 46 patients, 34 received corticosteroids (74%), of which 21 were responders (62%) and 13 non-responders (38%) (Supplementary Table S3). Non-treatment with corticosteroid was primarily due to infections (43%) or rapid clinical improvement (42%). Responders were more frequently young, non-obese men. Compared to non-responders, they had less hyperdynamic circulation (lower free hepatic vein pressure and B-type natriuretic peptide (NT-ProBNP) and lower systemic inflammation (VCAM, TNFRSF1A), despite similar (C-reactive protein, IL-6), MELD score, and HVPG (Table 2).

**Figure 4:**
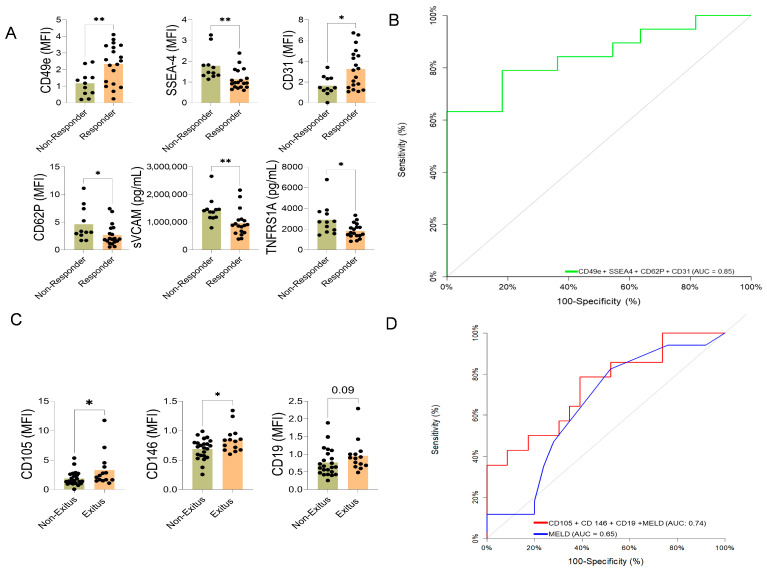
Extracellular vesicle (EV) surface antigen associated with corticosteroid response and long-term mortality in AH patients. (**A**) EV surface antigens associated with corticosteroid response and long-term mortality in AH patients. Comparison of EV surface antigen cargo between patients who responded to steroid treatment (responders) and those who did not (non-responders). Statistically significant differences were calculated using a t-test or Mann–Whitney based on normal or non-normal distribution * *p* < 0.05, ** *p* < 0.005. The black dots indicate the values for each patient individually. (**B**) Combined multivariate predictive model of corticosteroid response based on EV surface antigen. The diagonal gray line represents a classifier with no discriminative power (i.e., random chance). (**C**) Comparison of EV surface antigen expression between patients who survived (survivors) and those who did not survive (non-survivors) at long-term follow-up. The black dots indicate the values for each patient individually. (**D**) Receiver operating characteristic curve (ROC) curve comparison between the MELD score (red line) and a custom multivariate model (blue line) incorporating EV surface antigen markers for predicting long-term mortality. The EVs-based model outperforms the MELD score in predictive accuracy. Statistically significant differences were calculated using a *t*-test or Mann–Whitney based on normal distribution or non-normal distribution * *p* < 0.05.

Responders showed higher expression of EVs enriched with surface antigens CD49e and CD31, and lower levels of SSEA-4 and CD62p (Figure 4A).

Given the observed clinical differences between responders and non-responders, the limited number of events (21 responders), and the associated risk of model overfitting, we performed multivariable analyses with age and sex as the only covariates. Under this conservative approach, the EVs markers remained independently associated with corticosteroid response, including CD49e (OR = 3.14; 95% CI, 1.19–12.20; *p* = 0.045), CD31 (OR = 1.91; 95% CI, 1.02–3.91; *p* = 0.042), SSEA4 (OR = 0.056; 95% CI, 0.003–0.48; *p* = 0.025), and CD62p (OR = 0.58; 95% CI, 0.31–0.87; *p* = 0.030). These findings suggest that the associations between EV markers and corticosteroid response are not solely explained by age or sex, although residual confounding by other clinical factors cannot be fully excluded.

The individual association of each EVs antigens was evaluated by area under the curve (AUC), and the final corticosteroid response model included CD49e, CD31, SSEA-4, and CD62p with an AUC 0.847 (95% CI: 0.71–0.98, *p* < 0.001), and with a net reclassification improvement (NRI) (1.22 CI: (0.63–1.80), *p* < 0.001)—integrated discrimination improvement (IDI) (0.33 CI: (0.15–0.51), *p* < 0.001) (Figure 4B).

In addition, we assessed whether EVs could be associated with mortality during the median follow-up of 1.7 (0.64–2.70) years. Fifty-four percent of patients survived, whereas 37% died or underwent liver transplantation (LT), and 9% were lost to follow-up. Patients who died or underwent transplantation had a higher BMI, a more pronounced hyperdynamic circulation—characterized by higher free hepatic venous pressure and lower mean arterial pressure—and elevated IL-6 levels. The main characteristics of these patients are summarized and compared in Table 3.

These differences suggest a more severe inflammatory and circulatory profile in patients who died or underwent LT (Supplementary Table S4). The EVs associated with death/LT during follow-up showed increased expression of the B-cell marker CD19 and the angiogenesis-related molecules endoglin (CD105) and MCAM (CD146) (Figure 4C). The AUC evaluated the individual capacity of each EVs antigens (CD19, CD105, and CD146), and the final model included CD19, CD105, CD146, and MELD score with an AUC: 0.74 ((95% CI: 0.57–0.91), *p* = 0.006) vs. MELD score alone AUC: 0.65 ((95% CI: 0.48–0.82), *p* = 0.09), with an NRI (0.34 CI:(0.30–0.97), *p* = 0.29)—IDI (0.19 CI:(0.04–0.34), *p* = 0.015) (Figure 4D).

### 2.4. Impact of Alcohol Consumption During the First 90 Days After AH Epidose on EVs Surface Antigen Expression

At the 90-day follow-up, 30 patients (65%) were assessed for alcohol use through the AUDIT questionnaire and Phosphatidylethanol (PEth) levels. Of these, 57% maintained abstinence, while 43% continued drinking. In patients who resumed alcohol consumption (13 patients), EVs were enriched by monocyte (CD14) and B cell (CD20) markers (Supplementary Figure S5).

## 3. Discussion

To the best of our knowledge, this is the first study to characterize the enrichment of surface antigens on plasma EVs in AH using multiplex flow cytometry. We identified a significant increase in circulating EVs, enriched in surface antigens of lymphocyte activation, endothelial injury, and hepatic progenitor cell involvement (Supplementary Table S1). These EVs were associated with key clinical outcomes, including corticosteroid response and mortality. Key EVs enriched with antigens linked to corticosteroid response may have clinical utility, especially for non-responders identified on day four. This approach may support the timely selection of patients for early LT evaluation.

The first significant finding of this study is the quantitative and qualitative characterization of EVs in AH. Compared with HD, AH patients exhibited increased numbers of EVs enriched for markers of immune cells, endothelial cells, and liver progenitor cells. Most of these antigens, particularly those linked to monocyte and lymphocyte activation, may reflect the immunotoxic effects of alcohol and the possible underlying immune dysregulation in the coordination of EVs production. Notably, patients who continued alcohol consumption at 90 days showed persistent alterations in EVs enriched with immune cell markers, suggesting immune activation associated with subsequent alcohol relapse (Supplementary Figure S5). The increase in plasma EV quantity observed in AH patients is consistent with previous studies [16,27,28]. In addition, the expression of specific surface markers suggests that these EVs, enriched with dysregulated immune cells and endothelial markers, may contribute to an increased risk of bacterial infections during the AH episode, impaired recovery, and mediate the poor prognosis.

Nevertheless, our findings are consistent with a clinical study suggesting that EVs from alcohol-exposed hepatocytes, carrying liver-specific miR-122, sensitize monocytes to lipopolysaccharides and amplify inflammation [29]. Our data are consistent with a potential role of EVs-related signaling in immune dysregulation in AH; however, these findings are exploratory and may reflect complex interactions involving intrahepatic monocyte activity, endothelial activation, and liver progenitor cell responses rather than direct causal mechanisms [21,32,33]. Moreover, because our analysis was limited to circulating EVs, the precise cell and tissue origins of these vesicles remain to be confirmed.

The second major finding of this study is an increase in EVs enriched with antigens from the adaptive immune system and endothelial activation, both of which are associated with the two main clinical complications of AH during hospitalization: infections and AKI. EVs enriched with adaptive immune cell markers (e.g., reduced CD2-T lymphocyte activation and increased CD20-B lymphocyte activation) were linked to in-hospital infections, which aligns with previous evidence indicating that, during infectious diseases, immune cell-derived EVs carry antigen-presenting and activation molecules that modulate host immune responses [34]. In contrast, those enriched in endothelial (CD146) and immune activation (CD40) markers were associated with AKI. In addition, we observed a significant increase in inflammatory markers (IL-6, TNFRS1A, and sVCAM), which may be involved in the SIRS often observed in patients with AH [3]. To the best of our knowledge, this is the first report describing an increase in EVs bearing CD40 and CD146 in the setting of AKI. CD146 has been identified as a key player in the pathogenesis of inflammatory diseases and as a potential diagnostic marker for acute kidney transplant rejection [35]. In liver disease, CD146 has also shown promise as a cost-effective surrogate biomarker for cirrhosis, with a good correlation with disease severity [36]. Similarly, CD40 has been implicated in models predicting renal outcomes after LT, acting as an inflammatory mediator potentially involved in immune-mediated renal injury [37]. Given that endothelial dysfunction and microvascular injury are central mechanisms contributing to AKI, we hypothesize that peripheral EVs enriched in surface antigens of endothelial damage (CD146) and immune activation (CD40) may act as mediators of liver–kidney crosstalk. These vesicles may contribute to processes linked to kidney injury in AH, potentially through pathways involving endothelial activation, leukocyte recruitment, and tubular damage; however, these observations should be considered exploratory.

The third key finding is the identification of a specific plasmatic EVs antigen profile associated with corticosteroid response and long-term mortality in patients with AH. In this study, we demonstrated that the EV antigen profile in plasma can distinguish responders from non-responders to corticosteroid therapy. These EVs features may reflect a more favorable immune and endothelial microenvironment that supports therapeutic responsiveness. Responders were young, non-obese men with higher levels of EVs enriched with antigens of endothelial activation and cell transmigration, along with lower levels of liver progenitor cell antigens and inflammatory signals. To date, only one previous study has demonstrated the predictive value of EVs in this setting, showing that elevated levels of CD34+ (endothelial cells) and asialoglycoprotein receptor 1 (ASGPR1+) (expressed on the sinusoidal surface of hepatocytes) microvesicles may serve as non-invasive markers of non-response to corticosteroid [31]. However, our findings suggest that higher levels of EVs enriched in CD31 and CD49e, together with lower expression of SSEA4 and CD62p in responders, may be associated with enhanced, more organized cell migration, improved endothelial barrier integrity, and potentially contribute to increased responsiveness to corticosteroid therapy. In this context, EVs enriched mainly in CD31 may be involved in an early, adaptive, and reparative response that facilitates immune cell trafficking and tissue repair, thereby being associated with corticosteroid responsiveness [38]. Collectively, these EV-associated antigens linked to corticosteroid response may have clinical utility as early markers, particularly for identifying non-responders as early as day 4 of therapy. This approach may help support the timely identification of patients who could benefit from early liver transplantation evaluation. Nevertheless, these exploratory results and the hypothesis-generating nature of our findings require confirmation in future functional and prospective studies.

In contrast, elevated EVs expressing CD105, CD146, and CD19 were associated with increased mortality, suggesting sustained endothelial activation and B-cell dysregulation as adverse prognostic features. Antigens associated with increased mortality included antigens of adaptive immune activation (CD19) and endothelial activation (CD105 and CD146), as well as higher systemic inflammation (IL-6). These antigens have been studied individually in the context of liver disease. Elevated levels of IL-6 have been associated with poorer outcomes across various liver diseases [39]. CD19^+^ B cells, particularly intrahepatic memory B and plasma cell subpopulations, are altered in acute-on-chronic liver failure [40]. While direct studies evaluating CD105 and CD146 in the context of AH are currently lacking, our findings suggest that persistent or excessive endothelial activation over time may contribute to endothelial dysfunction, microvascular thrombosis, and subsequent poor clinical outcomes and increased mortality. In this context, EVs enriched in CD105 (endoglin), a transforming growth factor-β (TGF-β) co-receptor expressed on endothelial cells and involved in angiogenesis and vascular remodeling [41,42], may reflect heightened endothelial activation and proliferation. CD105 is a well-recognized marker of activated, proliferative endothelium and has been linked to pathological angiogenesis and fibrosis, correlating with disease severity and increased mortality. Altogether, these findings suggest that elevated EVs enriched in CD105 may indicate endothelial dysfunction and maladaptive angiogenic responses in severe disease [42,43]. CD146, as previously mentioned, has been proposed as a marker for cirrhosis and correlates with disease severity [36]. In our study, it has a strong positive correlation with the ABIC score. All these molecules may be relevant in liver disease progression and outcomes in AH. These changes likely reflect underlying pathogenic processes, including maladaptive endothelial activation and microvascular injury. Further studies are required to determine the clinical utility of these EV-associated antigens for risk stratification and transplant decision-making.

In summary, our findings suggest that EVs enriched with endothelial activation antigens may initially reflect an adaptive, reparative response associated with corticosteroid responsiveness, whereas persistent or excessive activation may lead to endothelial dysfunction and maladaptive processes, contributing to microvascular injury, inflammation, and multi-organ failure.

This study has several limitations: (a) a relatively small sample size from patients with AH; (b) the limited number of female patients included (which is a common feature of AH cohorts) may have limited the detection of potential sex-specific differences; (c) the absence of a group of patients admitted with ALD (limited generalizability to other stages of alcohol-associated liver disease; (d) one hospital center; and (e) the tissue origin of circulating EVs have yet to be fully elucidated. EVs expressing endothelial or immune-associated surface markers may predominantly reflect contributions from the corresponding cell types, although alternative cellular sources cannot be excluded. The strengths are as follows: the prospective design, systematic sampling and clinical characterization, the use of multiplex EV profiling technology, and the confirmation of abstinence via PEth at 90 days.

In conclusion, our findings demonstrate the potential clinical utility of profiling EV surface antigens in patients with AH. EVs enriched with surface markers of activated endothelial cells, adaptive immune cells, and hepatic progenitor cells were associated with significant clinical outcomes. The main EVs enriched with antigens associated with corticosteroid response could be applied in clinical practice, particularly for the early identification of non-responders on day four. This approach may support the timely selection of patients for early LT evaluation. These observations should be considered exploratory and hypothesis-generating. Further translational and functional research, along with validation in larger, multicenter cohorts, are warranted to confirm these observations and explore their application in guiding therapeutic decisions.

## 4. Materials and Methods

### 4.1. Population and Study Design

Consecutive patients with a clinical suspicion of AH were prospectively enrolled at Hospital de la Santa Creu i Sant Pau (Barcelona, Spain) between June 2020 and December 2024, before initiating corticosteroid treatment. All patients were followed prospectively until loss to follow-up, death, or LT. Clinical suspicion of AH was based on the following: excessive alcohol consumption (>60 g/day) before admission, moderately elevated aminotransferases (AST and ALT), high GGT, and serum bilirubin levels. Severe AH was defined as an Age, Bilirubin, INR, and Creatinine (ABIC) score ≥ 6.71 (ABIC B and C) or MEL > 20 points at admission, and histological confirmation was obtained for those patients on whom it was possible to perform a transjugular liver biopsy (TJLB) (36 patients) [9,44]. Psychiatric comorbidities were defined and classified according to DSM-5 [45]. The right cardiovascular pressures and HVPG were assessed according to the BAVENO VII guidelines [46].

Exclusion criteria were treatment with corticosteroids before TJLB, hepatocellular carcinoma, and active extra-hepatic cancer.

#### Blood Collection and Plasma Preparation

Peripheral blood was collected from 28 HD patients and from 46 AH patients at admission, using BD Vacutainer Citrate Tubes (BD Biosciences, San Jose, CA, USA). Platelet-poor plasma was obtained by centrifuging fresh whole blood at 1.000× *g* for 10 min. The resulting plasma was aliquoted and stored at −20 °C until analysis. According to the MISEV2018 guidelines and previous studies, EVs retain functional uptake characteristics after storage at −20 °C, supporting the suitability of this storage condition for EV analysis in clinical samples [24,25,47]. Blood cell counts and clinical and biochemical parameters were collected at baseline and at control times by hepatologists and the laboratory at Hospital de la Santa Creu i Sant Pau.

### 4.2. EVs Characterization

EVs’ size and concentration were assessed by NTA and visualized by Cryo-EM following MISEV 2024 guidelines [25].

#### 4.2.1. Nanoparticle Tracking Analysis (NTA)

The concentration and size distribution of the EV preparations were determined by measuring Brownian motion using a NanoSight LM10 system equipped with fast video capture and particle-tracking software v2.3.5 (Malvern Instruments, Malvern, UK). Pre- and post-acquisition settings were maintained the same for all samples, and each video was analyzed to determine the particle size mode and estimate particle concentration. Each sample was acquired three times. Then, an average curve was calculated for each sample.

#### 4.2.2. Cryo-Electron Microscopy

EVs preparations were directly adsorbed onto glow-discharged holey carbon grids R2/1 300 Mesh (QUANTIFOIL, Großlöbichau, Germany). Grids were blotted at 95% humidity and rapidly plunged into liquid ethane using a LEICA EM GP2 (Leica Microsystems, Wetzlar, Germany). Vitrified samples were imaged at liquid nitrogen temperature using a JEM1230 transmission cryo-electron microscope (JEOL, Tokyo Japan) equipped with a 120-kV LaB6 thermionic gun and with an Orius SC1000 (4008 × 2672 pixels) cooled slow-scan CCD camera (GATAN, Abingdon UK).

#### 4.2.3. EVs Surface Antigen Analysis by Multiplex Bead-Based Technique

The plasma samples were evaluated by a multiplex bead-based EVs capture and analyzed by flow cytometry using a MACSPlex EV kit IO, human [48]. Plasma samples were incubated with the beads overnight. There were 37 fluorescently labeled capture bead populations, each coated with a specific antibody binding the respective surface antigen, and two control bead populations. The EVs attached to the beads were identified using allophycocyanin-conjugated antibodies against CD9, CD63, and CD81. Median fluorescence intensity (MFI) was measured on a MACSQuant Analyzer 10 flow cytometer (Miltenyi, Bergisch Gladbach, Germany) in line with previous validation studies [49].

The assay was technically reproducible, and all final results were based on plasma-derived EVs. The stored plasma samples were processed using two consecutive centrifugations to further purify the plasma (30 min at 2000× *g*, 45 min at 10,000× *g*). A total of 30 µL of centrifuged plasma samples was diluted by 1/5 using the buffer solution, and the final volume added to each well of the kit was 130 µL of diluted plasma, which was analyzed. We used MacsPlex Buffer incubated with beads and detection antibodies as a blank control. All analyses were performed using normalized MFI (nMFI) values, calculated as the MFI of the individual marker divided by the MFI of its immunoglobulin isotype control, and sample evaluations were performed without knowledge of the clinical diagnosis. All antigens were analyzed simultaneously.

### 4.3. Determination of Soluble Pro-Inflammatory Mediators

Plasma concentrations of interleukin-6 (IL-6; catalog no. 3460-1H-6, Mabtech, Nacka Strand, Sweden), TNF receptor superfamily member 1A (TNFRSF1A; catalog no. DY225-05), vascular cell adhesion molecule-1 (VCAM-1; catalog no. DY809-05), and intercellular adhesion molecule-1 (ICAM-1; catalog no. DY720) were measured in patients with AH using commercially available ELISA kits and according to the manufacturers instructions (all from R&D Systems, Minneapolis, MN, USA).

### 4.4. Statistical Analysis

Normality of the data distribution was assessed using the Kolmogorov–Smirnov test. Continuous variables were expressed as mean (standard deviation) or median (interquartile range) as appropriate. Differences in continuous variables were tested by Student’s *t*-test, Mann–Whitney tests or the Wilcoxon signed rank test, considering independent or related samples as applicable. Categorical variables were presented as frequency and percentage. Differences in the categorical variables were assessed by the χ^2^ test or by Fisher’s exact test. Correlation between the Es antigens and quantitative variables was assessed using Spearman’s correlation. *p* < 0.05 were considered statistically significant. The association between the corticosteroid response and different EVs was analyzed using age- and sex-adjusted logistic regression models. Given their clinical relevance, the limited number of events, and the associated risk of model overfitting, the number of covariates included in multivariable analyses was intentionally restricted. To evaluate the predictive potential of EVs, antigens associated with clinical outcomes, such as corticosteroid response and survival, with *p*-values less than 0.1 in the initial descriptive analysis were selected for further assessment using the Area Under the Curve (AUC) and the DeLong test. Building upon a neutral baseline model, we sequentially examined the additional prognostic significance of each antigen and its combinations by measuring improvements in AUC, integrated discrimination improvement (IDI), and net reclassification improvement (NRI) [50]. To compare the progression of continuous variables between the two groups, linear mixed models were employed. All statistical analyses were conducted using R software (v.4.4) (R Foundation for Statistical Computing, Vienna, Austria), SPSS Statistics (v.29) (IBM Corp., Armonk, NY, USA), and GraphPad Prism (10.2.3) (GraphPad Software, San Diego, CA, USA).

## Figures and Tables

**Table 1:** Baseline demographic, clinical, and hemodynamic characteristics of patients with alcohol-associated hepatitis in the study cohort.

Characteristics	*n*	Patients (*n* = 46)
Baseline characteristics at admission		
Sex, Male/Female, (%)	46	35 (76%)/11 (24%)
Age, (y)	46	52.0 (44.0–58.0)
First episode of AH, (%)	46	35 (76%)
Alcohol intake, (g/day)	46	100.0 (100.0–150.0)
^a^ AUDIT score, (points)	39	22.0 (18.0–24.0)
^b^ Tobacco, (%)	46	35 (76%)
^c^ Psiquiatric comorbidities, (%)	46	20 (43.5%)
Cardiovascular comorbidity		
Diabetes, (%)	46	10 (22%)
Arterial hypertension, (%)	46	15 (33%)
Metabolic Syndrome, (%)	46	16 (35%)
BMI, Kg/m^2^	46	26.3 (24.4–31.5)
Obesity, (%)	46	14 (30%)
^d^ Overweight or Obesity, (%)	46	31 (67%)
^e^ Associated liver disease, (%)	46	20 (43%)
Stage of liver disease at admission		
Compensated, (%)	46	12 (26%)
First decompensation, (%)	46	19 (41%)
Recurrent decompensation, (%)	46	15 (33%)
Liver decompensation at admission		
Ascites, (%)	46	33 (72%)
Encephalopathy, (%)	46	11 (24%)
Variceal bleeding, (%)	46	5 (11%)
Laboratory data at admission		
Bilirubin, mg/dL	46	10.4 (5.3–14.1)
Creatinine, mg/dL	46	0.7 (0.6–0.8)
Prothrombin time, INR	46	1.8 (1.7–2.4)
AST, U/L	46	136.0 (92.5–172.0)
ALT, U/L	46	42.5 (30.0–65.2)
GGT, U/L	46	200.0 (128.7–495.5)
Albumin, g/L	46	24.3 (21.8–28.2)
Platelets, ×10^9^/L	46	86.5 (64.7–130.2)
Hemoglobin, g/L	46	106.5 (95.2–120.2)
Leukocyte count, ×10^9^/L	46	7.5 (6.0–10.9)
Neutrophil count, ×10^9^/L	46	5.3 (3.7–8.5)
Monocyte count, ×10^9^/L	46	0.8 (0.6–1.0)
Lymphocyte count, ×10^9^/L	46	1.0 (0.6–1.4)
Neutrophil-Lymphocyte Ratio	40	6.0 (2.9–9.4)
CRP, mg/L	45	29.6 (15.6–46.5)
IL-6, pg/mL	33	406.4 (123.0–1129.5)
AH severity scores		
MELD score	46	22.0 (20.0–24.0)
Maddrey score	46	46.2 (39.1–62.0)
ABIC score ABIC class (A/B/C)	46	8.1 (7.0–9.2) 6 (13%)/28 (61%)/12 (26%)
Child–Pugh score	46	11.5 (10.0–12.0)
Child–Pugh class (A/B/C), (%)	46	0 (0%)/8 (17%)/38 (83%)
Hemodynamic parameters at baseline		
^f^ HVPG, mmHg	40	20.0 (17.0–22.0)
Cardiac output, L/min/m^2^	36	8.7 (7.1–10.4)
Pulmonary artery pressure, mmHg	40	18.0 (14.0–21.0)
Pulmonary wedge pressure, mmHg	40	11.5 (7.0–15.7)
Right atrial pressure, mmHg	40	7.0 (5–9.7)
Systemic vascular resistance, mmHg	36	747.7 (606.7–964.9)
Mean arterial pressure, mmHg	46	88.8 (78.7–97.5)
Heart rate, beats/min	46	93.5 (84.5–104.2)
NT-proBNP, ng/L	22	171.0 (61.2–358.2)
Aldosterone, pmol/L	21	363.3 (183.9–777.2)
Plasma renin activity, µg/L/h	21	1.0 (0.2–3.2)

Data are presented for categorical variables as frequencies (%) and for continuous variables as median (Q1–Q3). ^a^ AUDIT: Alcohol Use Disorders Identification Test. ^b^ A total of 29 patients were active smokers (63%), 6 ex-smokers (13%). ^c^ Psychiatric comorbidities according to DSM-V (33): 13 (28%) patients had depressive plus anxiety disorder, 2 (4%) patients had depressive disorder alone, 4 (9%) patients had substance-related and addictive disorder, and 1 (2%) patient had depressive plus addictive disorder. ^d^ Overweight was defined as a BMI of 25–29.9, and obesity as a BMI ≥ 30. ^e^ The main associated liver diseases were as follows: 18 patients with metabolic-associated liver disease, 1 hepatitis-C associated virus, and 1 autoimmune-associated liver disease. ^f^ Hemodynamic study at baseline was performed in 40 patients. Only one patient had a previous Transjugular intrahepatic portosystemic shunt (TIPS). Abbreviations: ABIC, age-bilirubin-INR-creatinine score; AH, alcohol-associated hepatitis; AST, aspartate aminotransferase level; ALT, alanine aminotransferase level; AUDIT: Alcohol Use Disorders Identification Test; BMI: body mass index; CRP, C-reactive protein; GGT, γ-glutamyl transferase; HVPG, hepatic venous pressure gradient; MELD, model for end-stage liver disease; NT-proBNP, B-type natriuretic peptide.

**Table 2:** Baseline demographic, clinical, and hemodynamic characteristics of responder versus non-responder patients to corticosteroid *.

	AH Non-Responders (*n* = 13)	AH Responders (*n* = 21)	*p*
Baseline characteristics at admission			
Sex, Male/Female, (%)	6 (46%)/7 (54%)	17 (81%)/4 (19%)	0.005
Age, (y)	57.0 (51.5–64.5)	45.0 (40.0–56.5)	0.004
First episode of AH, (%)	10 (77%)	15 (71%)	0.740
Alcohol intake, (g/day)	100.0 (100.0–120.0)	120.0 (100.0–200.0)	0.193
^a^ AUDIT score, (points)	22.0 (18.0–23.0)	19.0 (15.0–24.5)	0.906
^b^ Tobacco, (%)	7 (54%)	17 (81%)	0.067
^c^ Psiquiatric comorbidities, (%)	5 (38.5%)	12 (57%)	0.183
Cardiovascular comorbidity			
Diabetes, (%)	4 (31%)	4 (19%)	0.640
Arterial hypertension, (%)	4 (31%)	7 (33%)	0.986
Metabolic Syndrome, (%)	7 (54%)	7 (33%)	0.147
BMI, Kg/m^2^	31.5 (24.1–34.4)	26.1 (24.2–31.2)	0.264
Obesity, (%)	8 (61.5%)	6 (29%)	0.004
^d^ Overweight or Obesity, (%)	10 (77%)	12 (57%)	0.395
^e^ Associated liver disease, (%)	10 (77%)	9 (43%)	0.116
Stage of liver disease at admission			
Compensated, (%)	1 (8%)	8 (38%)	0.054
First decompensation, (%)	5 (38.5%)	9 (43%)	
Recurrent decompensation, (%)	7 (54%)	4 (19%)	
Liver decompensation at admission			
Ascites, (%)	11 (85%)	13 (62%)	0.345
Encephalopathy, (%)	4 (31%)	5 (24%)	0.711
Variceal bleeding, (%)	1 (8%)	2 (9%)	0.744
Laboratory data at admission			
Bilirubin, mg/dL	12.0 (8.7–19.1)	11.7 (8.1–15.2)	0.958
Creatinine, mg/dL	0.8 (0.6–1.2)	0.6 (0.5–0.8)	0.151
Prothrombin time, INR	2.1 (1.7–2.9)	1.8 (1.6–2.2)	0.115
AST, U/L	127.0 (85.0–160.0)	154.0 (111.0–223.0)	0.119
ALT, U/L	36.0 (29.5–59.5)	47.0 (36.5–79.5)	0.215
GGT, U/L	137.0 (109.5–219.0)	349.0 (176.0–911.5)	0.017
Albumin, g/L	23.7 (21.9–27.0)	26.7 (23.1–30.6)	0.065
Platelets, ×10^9^/L	78.0 (67.0–89.5)	97.0 (58.5–158.5)	0.385
Hemoglobin, g/L	105.0 (81.0–126.0)	109.0 (100.5–122.0)	0.607
Leukocyte count, ×10^9^/L	6.2 (5.0–10.5)	7.9 (5.9–12.1)	0.348
Neutrophil count, ×10^9^/L	4.1 (3.2–8.1)	5.7 (3.6–9.6)	0.405
Monocyte count, ×10^9^/L	0.7 (0.4–1.0)	0.9 (0.7–1.1)	0.111
Lymphocyte count, ×10^9^/L	1.0 (0.5–1.3)	1.0 (0.7–1.4)	0.595
Neutrophil-Lymphocyte Ratio	6.1 (2.5–9.7)	4.4 (3.1–9.6)	0.950
CRP, mg/L	37.5 (17.6–77.9)	29.5 (15.7–39.8)	0.330
IL-6, pg/mL	521.1 (113.1–1139.7)	348.0 (132.8–1119.2)	0.775
AH severity scores			
Lille score day 4thº/day 7th	0.644 (0.455–0.848)/ 0.600 (0.565–0.870)	0.174 (0.083–0.372)/ 0.128 (0.051–0.290)	0.002 <0.001
MELD score	23.0 (20.5–28.0)	22.0 (20.0–23.5)	0.238
Maddrey score	61.0 (43.5–71.8)	46.0 (40.6–61.8)	0.215
ABIC score	9.2 (8.7–9.6)	7.6 (6.7–8.9)	0.002
Child–Pugh score	12.0 (11.0–13.5)	11.0 (8.5–12.0)	0.029
Child–Pugh class (A/B/C), (%)	0 (0%)/0 (0%)/13 (100%)	0 (0%)/6 (29%)/15 (71%)	0.034
Hemodynamic parameters at baseline			
HVPG, mmHg	19.9 (15.8–21.8)	20.0 (17.4–22.1)	0.719
Wedged hepatic venous, mmHg	37.5 (31–42.3)	32.3 (30.9–35.1)	0.122
Free hepatic venous, mmHg	18.0 (12.5–20)	13.0 (10.8–15.3)	0.026
Cardiac output, L/min/m^2^	8.7 (7.9–9.7)	9.0 (7.3–10.6)	0.505
Systemic vascular resistance, mmHg	686.7 (616.4–912.2)	644.2 (572.7–882.8)	0.698
Mean arterial pressure, mmHg	81.0 (72.5–94.0)	87.0 (76.5–100.0)	0.228
Heart rate, beats/min	90.0 (73.5–99.5)	95.0 (86.5–104.5)	0.123
NT-proBNP, ng/L	1237.0 (395.0–1392.0)	125.5 (68.8–233.3)	0.005

Data are presented for categorical variables as frequencies (%) and for continuous variables as median (Q1–Q3)1-Q3. Categorical variables were compared using the chi-squared test (*p*-value NS > 0.05). Continuous variables were compared using the non-parametric Mann–Whitney U test. The association between the corticosteroid response and different EVs was analyzed using age and sex-adjusted logistic regression models. * A total of 34 patients (74% of the cohort) received corticosteroid treatment. ^a^ AUDIT: Alcohol Use Disorders Identification Test. ^b^ Include active smokers and ex-smokers. In the non-responders group: six patients are active smokers, and one is an ex-smoker. In the Responders group, 16 patients were active smokers, and 1 was an ex-smoker. ^c^ Psychiatric comorbidities according to DSM-V (33): In the non-responders group, four patients had depressive plus anxiety disorder, and one patient had depressive disorder alone. In the Responders group: seven patients had depressive plus anxiety disorder, one patient had depressive disorder alone, three patients had substance-related and addictive disorder, and one patient had depressive plus addictive disorder. ^d^ Overweight was defined as a BMI of 25–29.9, and obesity as a BMI ≥ 30. ^e^ Associated liver disease: 17 metabolic-associated liver disease, 1 hepatitis-C-associated virus, 1 autoimmune-associated liver disease. Abbreviations: ABIC, age-bilirubin-INR-creatinine score; AH, alcohol-associated hepatitis; AST, aspartate aminotransferase level; ALT, alanine aminotransferase level; AUDIT: Alcohol Use Disorders Identification Test; BMI: body mass index; CRP, C-reactive protein; GGT, γ-glutamyltransferase; HVPG, hepatic venous pressure gradient; MELD, model for end-stage liver disease; NT-proBNP, B-type natriuretic peptide.

**Table 3:** Baseline demographic, clinical, and hemodynamic characteristics of patients Alive versus Liver Transplanted (LT) or Dead at long-term follow-up **.

	*n*	Patients Alive (*n* = 25)	Patients LT or Dead *** (*n* = 17)	*p*
Baseline characteristics at admission				
Sex, Male/Female, (%)	25/17	19 (76%)/6 (24%)	12 (71%)/5 (29%)	0.687
Age, (y)	25/17	52.0 (44.0–58.0)	55.0 (47.0–59.5)	0.376
First episode of AH, (%)	25/17	19 (76%)	13 (76%)	0.764
Alcohol intake, (g/day)	25/17	100.0 (100.0–130.0)	120.0 (100.0–175.0)	0.234
^a^ AUDIT score, (points)	24/13	22.5 (12.8–23.8)	22.0 (18.5–24.0)	0.511
^b^ Tobacco, (%)	25/17	19 (76%)	13 (76%)	0.764
^c^ Psiquiatric comorbidities, (%)	25/17	9 (36%)	10 (59%)	0.190
Cardiovascular comorbidity				
Diabetes, (%)	25/17	4 (16%)	5 (29%)	0.463
Arterial hypertension, (%)	25/17	8 (32%)	6 (35%)	0.654
Metabolic Syndrome, (%)	25/17	8 (32%)	6 (35%)	0.654
BMI, Kg/m^2^	25/17	25.6 (23.7–29.5)	28.3 (25.8–34.4)	0.032
Obesity, (%)	25/17	6 (24%)	7 (41%)	0.348
^d^ Overweight or Obesity, (%)	25/17	14 (56%)	14 (82%)	0.075
^e^ Associated liver disease, (%)	25/17	8 (32%)	9 (53%)	0.464
Stage of liver disease at admission				
Compensated liver disease, (%)	25/17	7 (28%)	4 (23%)	0.672
First decompensation, (%)	25/17	11 (44%)	6 (35%)	
Recurrent decompensation, (%)	25/17	7 (28%)	7 (41%)	
Liver decompensation at admission				
Ascites, (%)	25/17	17 (68%)	13 (77%)	0.599
Encephalopathy, (%)	25/17	7 (28%)	2 (12%)	0.096
Variceal bleeding, (%)	25/17	4 (16%)	1 (6%)	0.516
Laboratory data at admission				
Bilirubin, mg/dL	25/17	10.2 (4.9–12.9)	10.8 (6.2–17.3)	0.481
Creatinine, mg/dL	25/17	0.7 (0.6–0.8)	0.8 (0.6–1.2)	0.195
Prothrombin time, INR	25/17	1.8 (1.6–2.5)	1.9 (1.7–2.5)	0.626
AST, U/L	25/17	112.0 (84.5–164.0)	141.0 (112.0–171.0)	0.219
ALT, U/L	25/17	39.0 (29.5–71.5)	54.5 (31.7–61.7)	0.530
GGT, U/L	25/17	234.0 (125.5–521.0)	178.0 (127.5–292.5)	0.343
Albumin, g/L	25/17	24.4 (21.9–28.5)	23.7 (21.0–28.4)	0.377
Platelets, ×10^9^/L	25/17	87.0 (60.5–130.5)	86.0 (64.5–98.0)	0.626
Hemoglobin, g/L	25/17	102.0 (99.5–120.5)	113.0 (89.5–121.0)	0.817
Leukocyte count, ×10^9^/L	25/17	7.8 (6.1–11.5)	6.3 (4.7–9.7)	0.254
Neutrophil count, ×10^9^/L	25/17	5.4 (3.7–8.9)	4.1 (3.3–7.8)	0.299
Monocyte count, ×10^9^/L	25/17	0.8 (0.7–1.1)	0.7 (0.4–0.9)	0.069
Lymphocyte count, ×10^9^/L	25/17	1.1 (0.7–1.4)	0.8 (0.5–1.5)	0.254
Neutrophil-Lymphocyte Ratio	23/16	5.2 (2.8–8.9)	6.3 (2.7–10.4)	0.711
CRP, mg/L	25/17	20.5 (14.5–37.4)	38.0 (15.8–54.0)	0.260
AH severity scores				
MELD score	25/17	21.0 (20.0–23.5)	22.0 (21.0–24.0)	0.110
Maddrey score	25/17	42.7 (36.0–64.0)	48.6 (42.5–61.8)	0.282
ABIC score	25/17	8.0 (6.8–8.9)	8.8 (7.4–9.4)	0.249
Child–Pugh score	25/17	12.0 (10.0–13.0)	12.0 (10.0–12.0)	0.990
Child–Pugh class (A/B/C), (%)	25/17	0 (0%)/5 (20%)/20 (80%)	0 (0%)/2 (12%)/15 (88%)	0.677
Hemodynamic parameters at baseline				
HVPG, mmHg	22/15	20.0 (17.6–21.5)	22.0 (17.0–24.5)	0.213
HVPG ≥ 20 mmHg		13 (52%)	11 (65%)	0.214
Wedged hepatic venous, mmHg	21/15	32.5 (30.3–36.3)	36.0 (31.0–41.0)	0.109
Free hepatic venous, mmHg	21/15	12.0 (11.0–15.0)	16.5 (13.0–19.0)	0.025
Cardiac output, L/min/m^2^	20/13	8.7 (6.9–9.9)	8.9 (7.9–11.5)	0.224
Cardiac index, L/min/m^2^	20/13	4.5 (3.7–5.1)	4.5 (3.7–5.4)	0.768
Systemic vascular resistance, mmHg	20/13	693.2 (622.2–1031.6)	778.1 (561.3–925.6)	0.524
Mean arterial pressure, mmHg	25/17	89.0 (84.0–99.5)	79.0 (70.5–96.8)	0.048
Heart rate, beats/min	25/17	97.0 (79.0–107.0)	90.0 (84.0–101.0)	0.672
NT-proBNP, ng/L	13/8	116.0 (60.5–291.0)	303.0 (75.0–446.0)	0.385
Corticosteroid response, (%)	17/13	11 (44%)	6 (35%)	0.530

Data are presented for categorical variables as frequencies (%) and for continuous variables as median (Q1–Q3). Categorical variables were compared using the chi-squared test (*p*-value NS > 0.05). Continuous variables were compared using the nonparametric Mann–Whitney U test. ** A total of 42 patients completed the long-term follow-up, 1 patient lost follow-up during the first 90 days, and 3 patients did not complete the long-term follow-up (at the first year of follow-up). *** A total of 4 patients were Liver Transplanted, and 13 patients died. ^a^ AUDIT: Alcohol Use Disorders Identification Test. ^b^ Include active smokers and ex-smokers. In the Alive group, 15 patients are active smokers, and 4 are ex-smokers. In the LT or Dead group, 11 patients were active smokers, and 2 were ex-smokers. ^c^ Psychiatric comorbidities according to DSM-V (33): In the Alive group, there were seven patients who had depressive plus anxiety disorder, one patient with depressive disorder alone, and one patient with substance-related and addictive disorder. In the LT or Dead group: six patients had depressive plus anxiety disorder, one patient had depressive disorder alone, two patients had substance-related and addictive disorder, and one patient had depressive plus addictive disorder. ^d^ Overweight was defined as a BMI of 25–29.9, and obesity as a BMI ≥ 30. ^e^ Associated liver disease in Alive group of patients: eight metabolic-associated liver disease. In the Dead or LT group: 7 metabolic-associated liver disease, one hepatitis-C-associated virus, and one autoimmune-associated liver disease. Abbreviations: ABIC, age-bilirubin-INR-creatinine score; AH, alcohol-associated hepatitis; AST, aspartate aminotransferase level; ALT, alanine aminotransferase level; BMI: body mass index; CRP, C-reactive protein; GGT, γ-glutamyl transferase; HVPG, hepatic venous pressure gradient; MELD, model for end-stage liver disease; NT-proBNP, B-type natriuretic peptide.

## Data Availability

The data presented in this study are available from the corresponding author.

## Supplementary Materials

The following supporting information can be downloaded at: https://www.mdpi.com/article/10.3390/ijms27031258/s1.

## Abbreviations

The following abbreviations are used in this manuscript:

ABIC	Age-bilirubin-international normalized ratio-creatinine
AH	Alcohol-associated hepatitis
AKI	Acute Kidney injury
ALD	Alcohol-associated liver disease
ASGPR1	Asialoglycoprotein
AST	Aspartate aminotransferase
ALT	Alanine aminotransferase
AUC	Area under the curve
AUD	Alcohol use disorder
AUDIT	Alcohol use disorders identification test
BMI	Body mass index
CD146	Cluster of differentiation 146 (also known as MCAM)
DAMPs	Damage-associated molecular patterns
EVs	Extracellular vesicles
GGT	Gamma-glutamyl transpeptidase
HD	Healthy donors
HVPG	Hepatic venous pressure gradient
ICAM-1	Intercellular adhesion molecule 1
IDI	Integrated discrimination improvement
IL-6	Interleukin-6
LT	Liver transplantation
MELD	Model for End-Stage Liver Disease
MFI	Median fluorescence intensity
NRI	Net reclassification improvement
NTA	Nanoparticle tracking analysis
NT-proBNP	N-terminal pro-B-type natriuretic peptide
PEth	Phosphatidylethanol
S	soluble
SEC	Size-exclusion chromatography
SIRS	Systemic inflammatory response syndrome
TJLB	Transjugular liver biopsy
TNFRS1A	Tumor necrosis factor receptor superfamily member 1A
VCAM	Vascular cell adhesion molecule 1
VEGF-A	Vascular endothelial growth factor A
