# Supplementary material for: Immune and Endothelial-Related Extracellular Vesicles Are Associated with Corticosteroid Response and Mortality in Alcohol-Associated Hepatitis
Source: Int J Mol Sci. 2026 Jan 27;27(3):1258. doi: 10.3390/ijms27031258 (PMC12897947; doi:10.3390/ijms27031258)
Supplement: Supplementary file 1 [file ijms-27-01258-s001.zip › ijms-4091227-supplementary.pdf]

International Journal of
*Molecular Sciences*

MDPI

*Supplementary Material*

# Immune and Endothelial-Related Extracellular Vesicles Are Associated with Corticosteroid Response and Mortality in Alcohol-Associated Hepatitis

**Albert Guinart-Cuadra [1,2,3,†], Anna Brujats [2,4,†], Justyna Szafranska [5], Rubén Guerrero [6], Fernándo Dinamarca [7], Elisabet Cantó [1], Maria Poca [2,4], Eva Román [2,4], Elisabet Sánchez-Ardid [2], Javier Fajardo [4], Montserrat Camps [4], Maria Mulet [1], German Soriano [2,4], Àngels Escorsell [4], Juan M. Falcon-Perez [2,8,9], Esperanza Gonzalez [8], Andreu Ferrero-Gregori [4], Cristina Gely [4], Jorge Villalba [6], Ramón Bataller [10], Josepmaria Argemi [2,11,12,13,14], Rubén Osuna-Gómez [1,‡], Silvia Vidal [1] and Edilmar Alvarado-Tapias [1,2,4,*,‡]**

## Table of contents

## 1. Legends of supplementary tables

1.1. Table S1: Hypothesized cellular sources of circulating EVs in AH patients. ……………………………………..………2

1.2. Table S2: Clinical events during alcohol-associated hepatitis. ………………………………….……………..….…..…..3

1.3. Table S3: Baseline EVs antigen markers cargo of patients with alcohol-associated hepatitis responders versus non-responders to corticosteroid treatment. ……………...……………...…………………………………..………………………4

1.4. Table S4: Baseline EVs antigen markers of patients with alcohol-associated hepatitis alive versus dead or LT at long-term follow-up. ……………………………………………………………………………………………………….……6

## 2. Legends of supplementary figures

2.1. Figure S1: Flow chart depicting the study cohort of patients with alcohol-associated hepatitis (AH). ………………8

2.2. Figure S2: Morphological and quantitative analysis of plasma-derived extracellular vesicles in healthy donors and alcohol-associated hepatitis patients. ……………………………………………….………...………………………………9

2.3. Figure S3: Correlation matrix of surface antigen cargo on EVs from healthy donors. …………………………..……10

2.4. Figure S4: Correlation between EVs surface marker cargo expression and clinical severity scores in AH Patients. ……………………………………………………………………………………………………………………………11

2.5. Figure S5: Longitudinal analysis of EVs surface marker expression in AH patients over 90Days. ……....................12

**Table S1.** Hypothesized cellular sources of circulating EVs in AH patients.

| | CD4 T cells | CD8 T cells | Natural Killers | Monocytes Macro-phages | Granulo-cytes | Plate-lets | Endothelial cells | Epithelial Cells | Stem Cells |
|---|---|---|---|---|---|---|---|---|---|
| CD146 | | | | | | | X | | |
| SSEA-4 | | | | | | | | | X |
| CD14 | | | | X | | | | | |
| CD105 | | | | | | | X | | |
| CD133 | | | | | | | | X | |
| CD326 | | | | | | | | X | |
| CD62P | | | | | | | X | | |
| CD209 | | | | | | | | | |
| CD2 | X | | X | | | | | | |
| CD44 | | | | X | | | | | |
| CD45 | X | X | X | X | X | | | | |
| CD25 | X | X | X | X | X | | | | |
| CD40 | | X | | X | | | | | |
| CD3 | X | X | | | | | | | |
| CD49e | | | | | | | X | | |
| HLA-ABC | X | X | X | X | X | | X | X | X |
| CD31 | | | | | | | X | | |
| CD42a | | | | | | X | | | |
| CD29 | X | X | X | X | X | X | X | X | X |
| CD41b | | | | | | X | | | |

Surface antigens of extracellular vesicles (EVs) are listed in the first column. Antigens highlighted in red indicate higher expression in patients with alcohol-associated hepatitis (AH) compared to healthy donors, whereas those highlighted in green indicate lower expression in AH patients. An "X" denotes that EVs carrying the corresponding surface antigen can potentially originate from the cell type indicated in the column header.

**Table S2.** Clinical events during alcohol-associated hepatitis admission.

| Events | Patients ($n$=46) |
|---|---|
| Infection, (%) | 20 (43%) |
| Acute Kidney Injury, (%) | 12 (26%) |
| [a]ICU, (%) | 5 (11%) |
| [b]OIT, (%) | 4 (9%) |
| Withdrawal Syndrome, (%) | 13 (28%) |
| Liver decompensation | |
| Ascites, (%) | |
| Onset during hospitalization | 18 (39%) |
| Worsening of previous | 14 (30%) |
| Hepatic encephalopathy, (%) | 19 (41%) |
| Variceal bleeding, (%) | 4 (9%) |
| Death during admission | 1 (2%) |

Data for categorical variables are presented as frequencies (%). a: Intensive Care Unit admission; b: Orotracheal intubation.

**Table S3.** Baseline extracellular vesicles (EVs) antigen markers of patients with alcohol-associated hepatitis (AH) responders versus non-responders to corticosteroid treatment.

| | ***n*** | AH Non-Responders (*n*=13) | AH Responders (*n*=21**)** | *p* |
|---|---|---|---|---|
| CD19 | 41 | 0.9 (0.6 - 1.0) | 0.8 (0.6 - 1.0) | 0.689 |
| CD4 | 41 | 0.4 (0.3 - 0.7) | 0.5 (0.4 - 0.6) | 0.662 |
| CD3 | 41 | 1.0 (0.5 – 1.8) | 1.9 (0.9 - 2.8) | 0.222 |
| CD105 | 41 | 2.8 (1.7 - 4.4) | 1.5 (1.3 - 2.5) | 0.401 |
| CD56 | 40 | 2.1 (1.3 - 3.1) | 1.5 (1.4 - 2.5) | 0.602 |
| HLA_DRD | 36 | 3.6 (2.3 - 4.3) | 2.8 (2.2 - 3.6) | 0.681 |
| CD8 | 41 | 1.9 (1.4 - 3.3) | 1.9 (1.3 - 3.5) | 0.815 |
| Ror1 | 41 | 1.0 (0.7 – 2.9) | 1.3 (0.9 - 2.2) | 0.720 |
| CD49e | 41 | 1.2 (0.6 - 1.5) | 2.5 (1.2 - 3.4) | 0.003 |
| CD25 | 41 | 0.9 (0.6 - 1.2) | 1.2 (1.1 - 1.7) | 0.619 |
| CD1c | 41 | 0.4 (0.4 - 0.9) | 0.5 (0.4 - 0.6) | 0.462 |
| CD2 | 41 | 0.6 (0.4 - 0.7) | 0.6 (0.5 - 0.9) | 0.356 |
| CD40 | 41 | 1.0 (0.9 - 1.2) | 1.2 (1.1 - 1.5) | 0.136 |
| CD63 | 41 | 6.9 (4.2 – 10.2) | 10.6 (7.6 - 15.5) | 0.368 |
| Hla_abc | 41 | 2.3 (1.9 - 3.4) | 2.7 (1.6 – 3.9) | 0.573 |
| Ssea4 | 41 | 1.5 (1.3 - 1.8) | 1.0 (0.8 - 1.4) | 0.017 |
| CD9 | 41 | 3.5 (2.7 – 6.7) | 5.0 (4.2 – 10.5) | 0.190 |
| CD209 | 41 | 0.4 (0.4 - 0.6) | 0.5 (0.4 - 0.6) | 0.519 |
| CD41b | 41 | 4.2 (3.0 - 5.4) | 5.2 (4.0 - 6.9) | 0.405 |
| CD146 | 41 | 0.7 (0.6 - 0.8) | 0.8 (0.6 - 0.9) | 0.349 |
| Mcsp | 40 | 1.1 (0.8 - 1.2) | 0.8 (0.8 - 1.1) | 0.385 |
| CD81 | 41 | 8.0 (5.4 - 23.7) | 11.0 (6.0 - 15.0) | 0.670 |
| CD11c | 41 | 0.7 (0.4 – 0.9) | 0.7 (0.6 - 0.9) | 0.299 |
| CD62p | 40 | 3.2 (2.8 – 6.3) | 2.0 (1.5 - 3.4) | 0.075 |
| CD133 | 41 | 1.5 (1.1 - 2.4) | 2.4 (1.3 - 3.7) | 0.191 |
| CD326 | 41 | 2.3 (1.2 – 3.8) | 1.7 (1.4 - 2.7) | 0.605 |
| CD44 | 41 | 0.9 (0.9 - 1.0) | 1.1 (0.9 - 1.3) | 0.201 |
| CD86 | 41 | 0.6 (0.5 - 0.7) | 0.7 (0.6 - 0.9) | 0.456 |
| CD24 | 41 | 0.8 (0.7 - 1.0) | 1.0 (0.8 - 1.5) | 0.099 |
| CD42a | 41 | 2.1 (1.4 - 4.0) | 2.2 (1.5 - 3.6) | 0.951 |
| CD31 | 41 | 1.4 (1.2 - 2.3) | 3.1 (1.6 - 4.6) | 0.004 |

| | | | | |
|---|---|---|---|---|
| CD45 | 41 | 0.9 (0.7 - 1.1) | 1.0 (0.9 - 1.4) | 0.337 |
| CD142 | 41 | 0.8 (0.8 - 1.0) | 0.9 (0.8 - 1.0) | 0.425 |
| CD69 | 41 | 1.3 (0.9 – 2.7) | 1.2 (0.9 - 2.1) | 0.694 |
| CD29 | 41 | 2.3 (1.4 - 7.5) | 6.2 (3.0 – 8.9) | 0.718 |
| CD14 | 41 | 1.2 (0.6 - 2.4) | 1.2 (0.9 - 1.4) | 0.537 |
| CD20 | 41 | 1.0 (0.8 - 1.7) | 0.8 (0.7 - 1.0) | 0.065 |
| Inflammatory markers | | | | |
| CD40L | 32 | 1257.3 (744.6 – 1527.3) | 1049.1 (541.8 - 1718.2) | 0.879 |
| CD62p | 32 | 70847.1 (41122.3 – 78141.3) | 49218.6 (33829.6 – 80620.9) | 0.344 |
| CD54 | 40 | 572854.3 (417859.3 – 1097599.8) | 492191.1 (262290.6 – 1094453.8) | 0.518 |
| CD106 (sVCAM) | 40 | 1392938.8 (1170796.6 – 1465599.1) | 885681.9 (618725.3 – 1088194.0) | 0.004 |
| TNRFS1a | 40 | 2695.3 (1781.0 – 3634.0) | 1636.4 (1366.6 – 2270.6) | 0.010 |
| Zonulin | 36 | 32.3 (11.3 – 70.9) | 42.4 (15.2 – 132.8) | 0.517 |
| IL-6, pg/mL | 33 | 521.1 (113.1 – 1139.7) | 348.0 (132.8 – 1119.2) | 0.799 |

Data are presented as median + quartiles Q1-Q3. Variables were compared using the nonparametric Mann-Whitney U test. P-value NS >0.10. Abbreviations: Alcohol-associated hepatitis (AH), Cluster differentiation (CD), Interleukin 6 (IL-6), tumor necrosis factor receptor superfamily member 1ª( TNRFS1a), soluble vascular cell adhesion molecule (sVCAM-1).

**Table S4.** Baseline extracellular vesicles (EVs) antigen markers of patients with alcohol-associated hepatitis alive versus dead or LT at long-term follow-up.

| | *n* | Patients alive (*n*=25) | Patients dead or liver transplantation (*n*=17) | ***p*** |
|---|---|---|---|---|
| CD19 | 41 | 0.6 (0.5 - 0.9) | 0.9 (0.7 - 1.0) | 0.091 |
| CD4 | 41 | 0.4 (0.4 - 0.6) | 0.4 (0.4 - 0.6) | 0.976 |
| CD3 | 41 | 1.3 (0.7 - 2.2) | 1.9 (0.9 - 2.6) | 0.203 |
| CD105 | 41 | 1.4 (1.1 - 2.5) | 2.2 (1.6 - 3.3) | 0.029 |
| CD56 | 40 | 1.5 (1.2 - 2.1) | 2.2 (1.6 - 3.3) | 0.113 |
| HLA_DRD | 36 | 3.0 (2.2 - 4.2) | 2.8 (2.4 - 3.7) | 0.972 |
| CD8 | 41 | 2.3 (1.2 - 3.4) | 2.0 (1.3 - 2.8) | 0.371 |
| Ror1 | 41 | 1.1 (0.8 – 2.2) | 1.3 (0.8 – 1.7) | 1.000 |
| CD49e | 41 | 1.7 (1.0 - 2.5) | 1.7 (1.1 - 2.4) | 0.951 |
| CD25 | 41 | 1.0 (0.6 - 1.2) | 1.0 (0.8 - 1.7) | 0.293 |
| CD1c | 41 | 0.4 (0.4 - 0.6) | 0.5 (0.4 - 0.7) | 0.215 |
| CD2 | 41 | 0.5 (0.4 - 0.6) | 0.6 (0.4 - 0.7) | 0.227 |
| CD40 | 41 | 1.1 (0.9 - 1.3) | 1.2 (1.0 - 1.3) | 0.667 |
| CD63 | 41 | 8.3 (5.8 - 11.6) | 8.1 (2.7 - 10.6) | 0.713 |
| Hla_abc | 41 | 2.7 (1.8 - 3.5) | 2.2 (1.8 - 3.3) | 0.580 |
| Ssea4 | 41 | 1.3 (0.8 - 1.8) | 1.2 (1.0 - 1.5) | 0.927 |
| CD9 | 41 | 5.8 (4.2 - 9.1) | 3.5 (2.4 - 5.5) | 0.059 |
| CD209 | 41 | 0.4 (0.4 - 0.5) | 0.4 (0.4 - 0.6) | 0.451 |
| CD41b | 41 | 5.2 (3.6 - 7.2) | 4.6 (3.0 - 5.4) | 0.279 |
| CD146 | 41 | 0.7 (0.6 - 0.8) | 0.8 (0.7 - 0.9) | 0.045 |
| Mcsp | 40 | 0.8 (0.7 - 1.0) | 0.9 (0.8 - 1.2) | 0.138 |
| CD81 | 41 | 11.4 (6.9 – 16.2) | 7.4 (5.6 - 13.4) | 0.279 |
| CD11c | 41 | 0.6 (0.4 - 0.8) | 0.7 (0.5 - 0.8) | 0.322 |
| CD62p | 40 | 3.6 (1.6 - 5.9) | 2.7 (1.8 - 3.2) | 0.322 |
| CD133 | 41 | 1.4 (1.1 - 2.7) | 1.5 (1.2 - 2.4) | 0.830 |
| CD326 | 41 | 1.6 (1.2 - 3.1) | 1.6 (1.2 - 2.4) | 0.899 |
| CD44 | 41 | 0.9 (0.9 - 1.1) | 1.1 (1.0 - 1.1) | 0.239 |
| CD86 | 41 | 0.6 (0.6 - 0.9) | 0.7 (0.6 - 0.8) | 0.782 |
| CD24 | 41 | 1.0 (0.6 - 1.5) | 1.0 (0.7 - 1.4) | 0.713 |
| CD42a | 41 | 2.8 (1.5 - 4.2) | 2.2 (1.9 - 3.2) | 0.460 |
| CD31 | 41 | 1.8 (1.3 - 3.4) | 2.2 (1.4 - 2.5) | 0.782 |

| | | | | |
|---|---|---|---|---|
| CD45 | 41 | 1.0 (0.8 - 1.1) | 1.0 (0.9 - 1.4) | 0.479 |
| CD142 | 41 | 0.8 (0.8 - 0.9) | 0.8 (0.8 – 0.9) | 0.988 |
| CD69 | 41 | 1.2 (0.8 - 2.1) | 1.2 (0.9 – 1.9) | 0.854 |
| CD29 | 41 | 4.4 (2.2 - 7.4) | 3.0 (1.8 – 7.4) | 0.736 |
| CD14 | 41 | 1.2 (0.9 - 1.6) | 1.0 (0.7 - 1.3) | 0.215 |
| CD20 | 41 | 0.9 (0.8 - 1.2) | 0.8 (0.8 - 1.1) | 0.499 |
| Inflammatory markers. | | | | |
| CD40L | 32 | 992.7 (286.4 – 1493.6) | 1156.4 (597.8 – 1886.6) | 0.370 |
| CD62p | 34 | 50824.2 (35831.5 – 70079.8) | 74160.9 (37814.5 – 83861.6) | 0.228 |
| CD54 | 40 | 428015.9 (263685.2 – 842228.4) | 459018.9 (408673.2 – 1009700.7) | 0.309 |
| CD106 (sVCAM) | 40 | 956014.9 (662984.1 – 1163989.9) | 1052624.6 (822845.4 – 1454214.2) | 0.352 |
| TNRFS1a | 40 | 1715.7 (1352.0 – 2173.2) | 2257.9 (1537.7 – 2764.6) | 0.058 |
| Zonulina | 36 | 25.7 (17.4 – 93.7) | 37.5 (23.8 – 73.7) | 0.302 |
| IL-6, pg/mL | 33 | 228.5 (108.1 – 521.1) | 987.5 (276.3 – 1602.4) | 0.027 |

Data are presented as median + quartiles Q1–Q3. Variables were compared using the nonparametric Mann-Whitney U test. P-value NS > 0.10. Abbreviations: Alcohol-associated hepatitis (AH), Cluster differentiation (CD), Interleukin 6 (IL-6), tumor necrosis factor receptor superfamily member 1 (TNRFS1a), soluble vascular cell adhesion molecule (sVCAM-1).

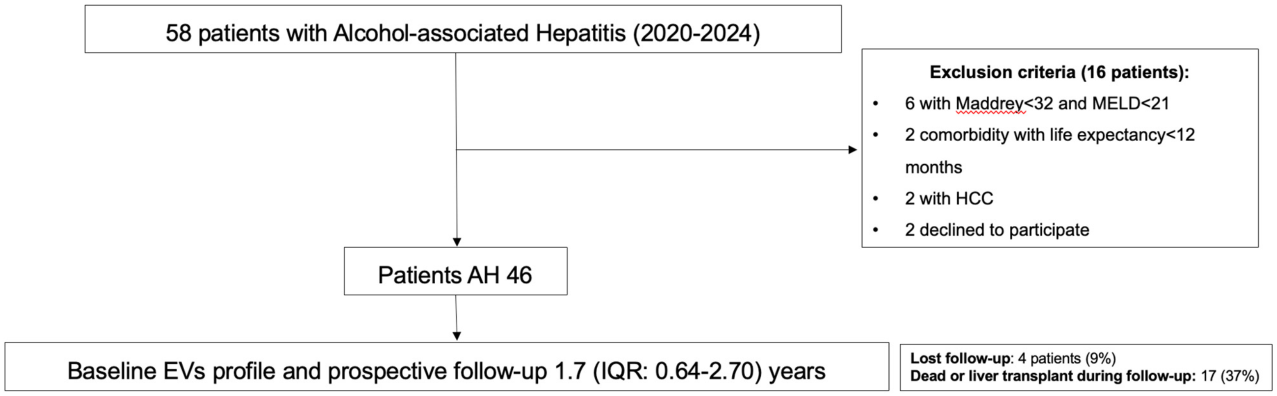


**Figure S1.** Flow chart depicting the study cohort of patients with alcohol-associated hepatitis (AH).

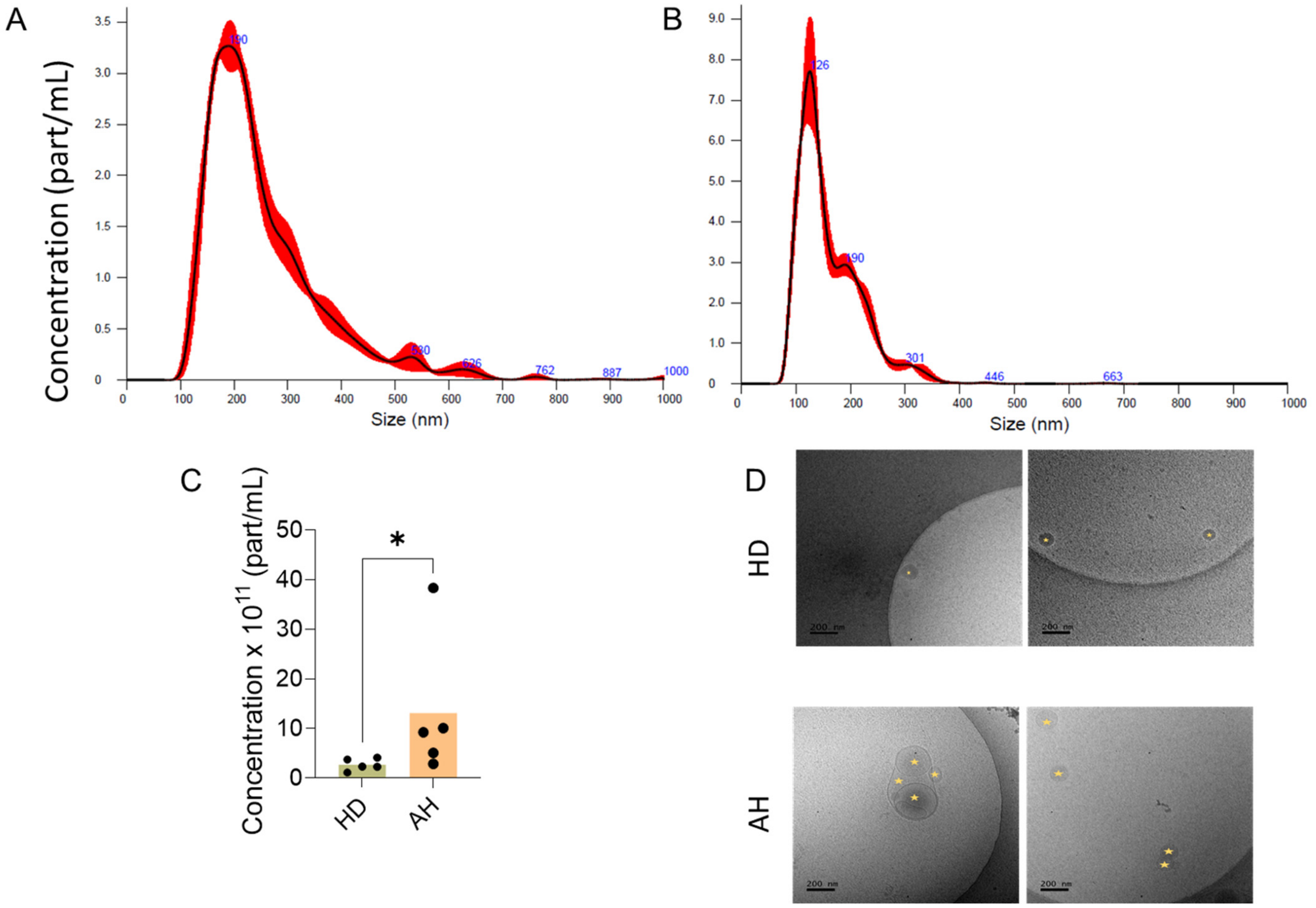


**Figure S2.** A) Size distribution and concentration report obtained from processing by Nanoparticle Tracking Analysis (NTA), A) AH extracellular vesicles (EVs) size distribution, B) HD EVs size distribution. C) Particle concentration determined by NTA in a subgroup of HD versus patients with AH, analyzed using the Mann-Whitney test (*$p<0.05$). The black dots indicate the values for each patient individually.D) Cryo-electron microscopy (cryo-EM) images of EVs derived from HD and AH patients. Representative images showing the morphology of small EVs isolated from plasma samples of HD and AH patients. EVs are highlighted with yellow stars for clarity.

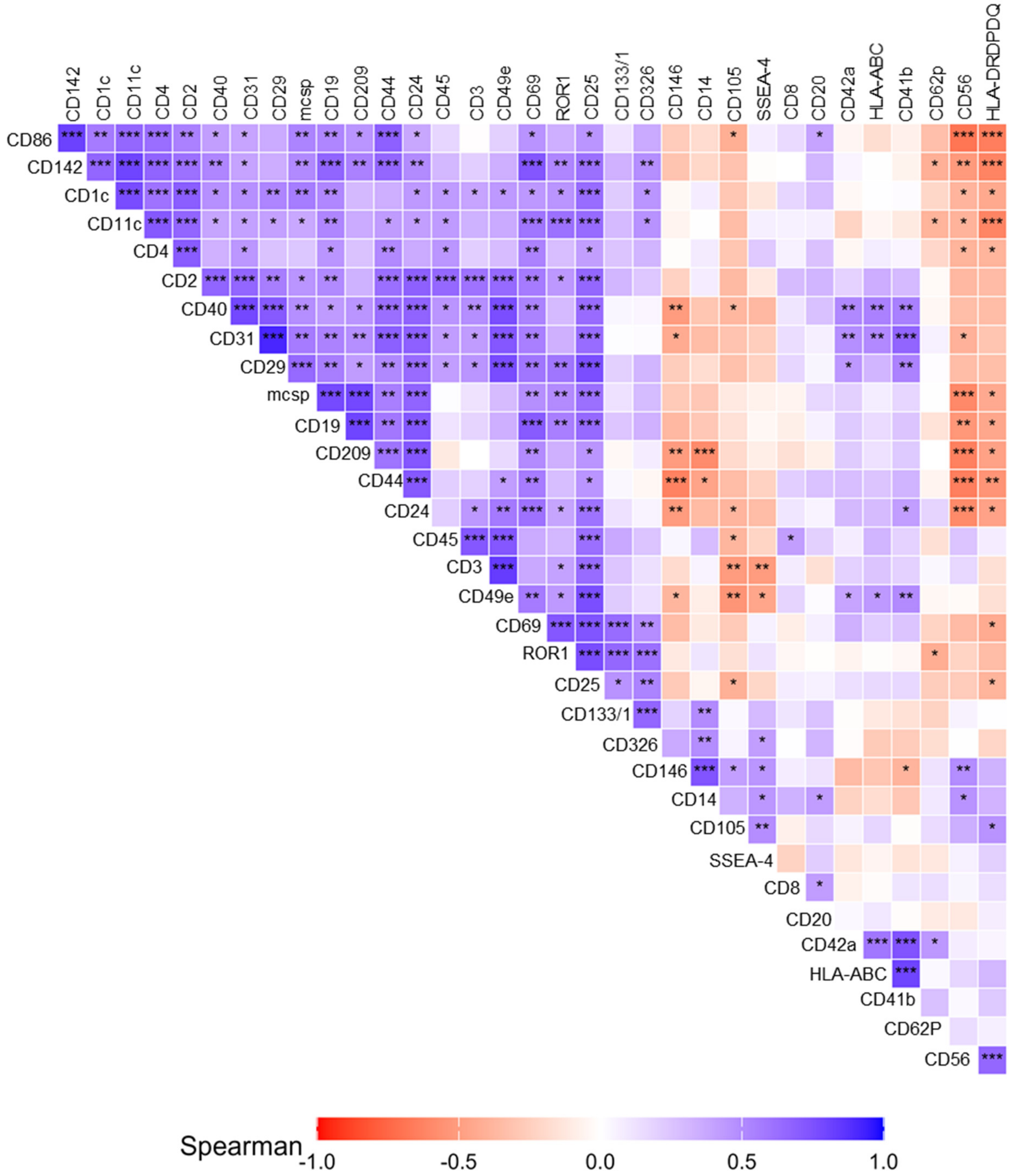


**Figure S3.** Correlation Matrix of surface antigens expressed on extracellular vesicles (EVs) from Healthy donors. The heatmap displays pairwise Spearman correlation coefficients, with positive correlations shown in blue and negative correlations in red. * $p < 0.05$, ** $p < 0.01$, *** $p < 0.001$.

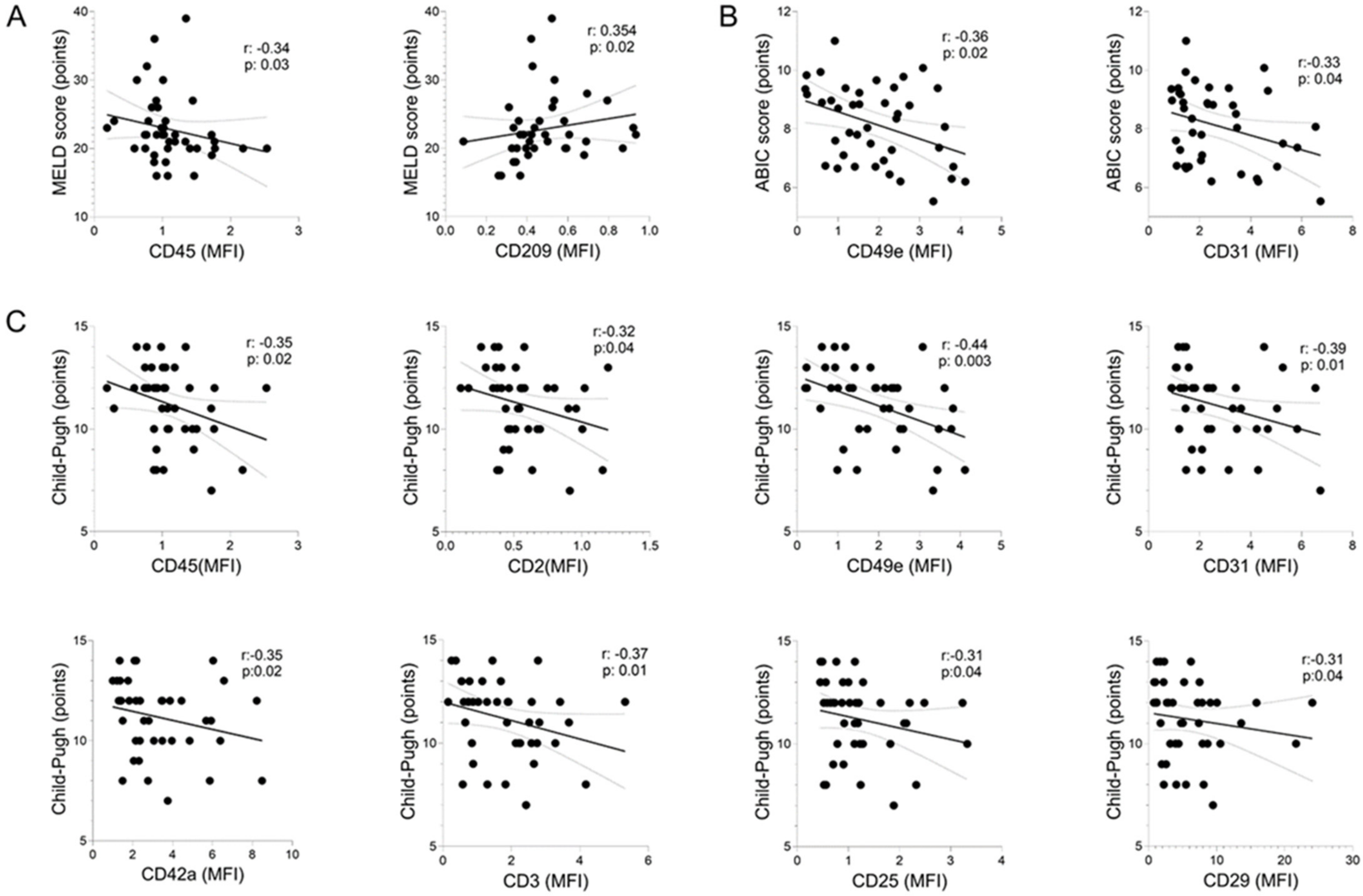


**Figure S4.** Correlations between the surface marker-enriched extracellular vesicles (EVs) and clinical severity score in alcohol-associated hepatitis (AH) patients. A) Correlation between EVs surface antigens expression and MELD score. B) Correlation between EVs surface antigens expression and ABIC, and C) Correlation between EVs surface marker expression and Child-Pugh score.

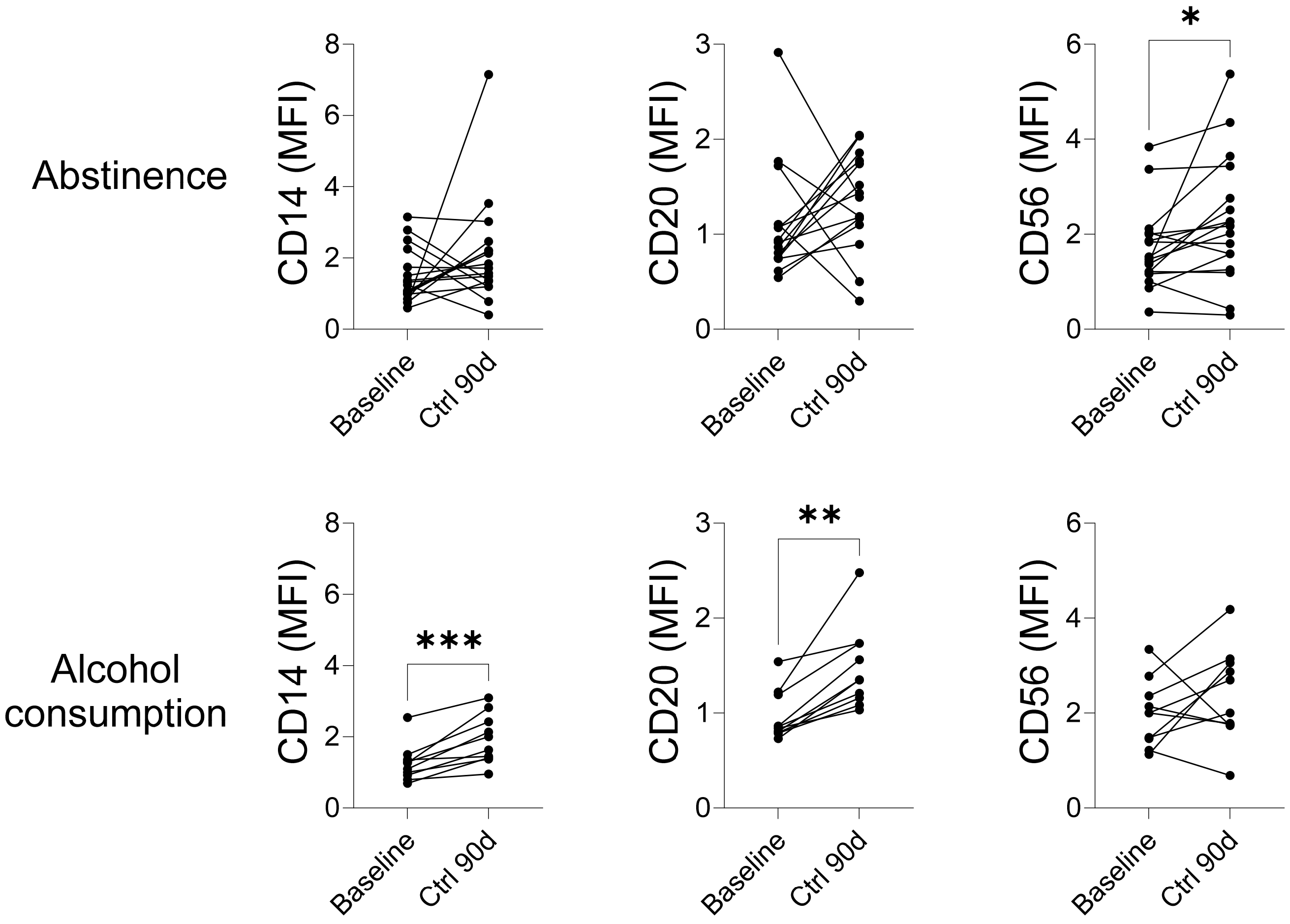


**Figure S5.** Follow-up analysis of extracellular vesicles (EVs) markers in alcohol-associated hepatitis (AH) patients. Differences in EVs markers between Baseline and 90-day control (Ctrl 90d) in patients who maintained abstinence versus those who continued alcohol consumption. Comparisons were performed using paired *t-tests* or Wilcoxon signed-rank tests as appropriate. *$p<0.05$, ** $p<0.005$, ***$p<0.001$.
